# Contemporary practices of physical trainers in professional soccer: A qualitative study

**DOI:** 10.3389/fpsyg.2023.1101958

**Published:** 2023-09-19

**Authors:** Joel Barrera-Díaz, António J. Figueiredo, Adam Field, Bruno Ferreira, Sérgio M. Querido, João Renato Silva, João Ribeiro, Ismael Pinto, Patricio Cornejo, Hernan Torres, Alvaro Saffa, Hugo Sarmento

**Affiliations:** ^1^University of Coimbra, Research Unit for Sport and Physical Activity, Faculty of Sport Sciences and Physical Education, Coimbra, Portugal; ^2^Department of Sport and Exercise Science, Research Centre for Musculoskeletal Science and Sports Medicine, Manchester Metropolitan University, Manchester, United Kingdom; ^3^Sports Science School of Rio Maior, Polytechnic Institute of Santarém, Rio Maior, Portugal; ^4^Faculty of Human Kinetics, University of Lisbon, Cruz Quebrada-Dafundo, Portugal; ^5^Center of Research, Education, Innovation and Intervention in Sport (CIFI2D), University of Porto, Porto, Portugal; ^6^Research Center in Sports Sciences, Health Sciences and Human Development (CIDESD), University of Maia (ISMAI), Maia, Portugal; ^7^Department of Performance Optimization, Gabinete de otimização desportiva, Sporting Clube de Braga SAD, Braga, Portugal; ^8^Athletic Club Barnechea, Santiago, Chile; ^9^San Luis de Quillota, Quillota, Chile; ^10^Club de Deportes Antofagasta, Antofagasta, Chile; ^11^Universidad Andrés Bello, Dirección Nacional de Deportes, Santiago, Chile

**Keywords:** physical abilities, evaluation, monitoring and control, injury prevention, optimization, professional football

## Abstract

**Introduction:**

Physical trainers (PTs) are integral for managing load, reducing injury and optimizing performance in professional soccer. However, little is known about how this practitioners operate in the applied setting and how some of the nuances experienced influence practice.

**Methods:**

This study explored the contemporary practices of PTs in professional soccer. Semi-structured interviews were undertaken with eight PTs from different professional teams in European and South American leagues. Interview questions were designed to extract information on the evaluation of physical abilities, monitoring and control of training and injury prevention. Subsequently, the interviews were video-recorded, transcribed, translated and analyzed using a content analysis approach.

**Results:**

The results suggest that the evaluation of physical capacities is carried out by PTs at the beginning of the preseason. It also appears that it is attempted that this process of regular testing is applied during the competitive period, with most participants conducting partial physiological and physical evaluations at different stages throughout the competitive season. In relation to the monitoring and control of training, subjective feedback scales are used to estimate the internal load, and the use of GPS devices is common to quantify external loads. Injury prevention programmes were implemented by all participants and were generally in a multi-component format focused on preventing or optimizing physical capabilities.

**Discussion:**

These insights can be used as a scientific reference point to inform applied practice in professional soccer, especially for practitioners that are inexperienced and aspiring to enhance how they operate in the field. Future investigations should explore the practices of PTs in detail and across a wider network in order to gain deeper and comprehensive insights into the applied soccer environment.

## 1. Introduction

Soccer is characterized as a high-intensity, intermittent team sport with many games across a competitive season (Turner and Stewart, 2014; Konefal et al., 2018). Professional soccer physical trainers (PTs) must use their understanding and experience of sports science and training to optimize physical and athletic performance while reducing injury risk (Weldon et al., 2021). Such expert sports science support ensures professional soccer players continue to develop and maintain a high level of conditioning during the season (Turner and Stewart, 2014). It is key that this process is optimized to enhance performance (Bangsbo, 2014; Turner and Stewart, 2014; Slimani and Nikolaidis, 2017; Ribeiro et al., 2021); however, there is less research to facilitate the understanding of how PTs operate within the applied environment. Contemporary research has surveyed strengths and conditioning practitioners (Weldon et al., 2021); however, this research mainly included European practitioners and used survey methods with critics arguing that insights lack key details that might be captured during interviews (Bailer, 2014). Therefore, it appears warranted to obtain a deeper understanding of applied practice across several continents to design and implement periodized training plans (Bompa and Buzzichelli, 2019).

The role of the PTs is complex and multifaceted, with the role including the development and maintenance of strength and endurance capabilities for optimal performance (Jones et al., 2013). The planning involved across an entire season includes optimizing both performance and recovery (Impellizzeri et al., 2004; Gaudino et al., 2015b; Moalla et al., 2016), in addition to ensuring an ideal workload to minimize injuries (Jaspers et al., 2017). To achieve this balance, adaptations to the volume and intensity of training must be made around matches, facilitating harmony between adaptations and recovery (Springham et al., 2018). It may also be necessary to individualize training loads due to the differences in individual training history, physical condition, body composition, strength asymmetries, and injury history (Bahr and Tron, 2005). Different internal and external load monitoring tools are commonly used both in training and competition (Newton et al., 2019). To estimate the internal load, tools such as the subjective perception of effort are currently used (Gabbett and Whiteley, 2017), and objective measures of exercise intensity, such as autonomic heart rate regulation indices (exercise heart rate) (Ali and Farrally, 1991) or biochemical markers (creatine kinase [CK]) (Lazarim et al., 2009a; Ribeiro et al., 2022), are also currently used. For external load, the most used tools are tracking systems (global positioning systems [GPS]) (Weldon et al., 2021), and these parameters provide information on work performed by each individual player. This information allows the PTs in professional soccer to make informed decisions regarding the timing of training sessions (Newton et al., 2019). However, it is precisely unclear how these methods are adopted to inform periodization and strength and conditioning programs.

Injury prevalence is high in professional soccer (Pérez-Gómez et al., 2020) and has a detrimental economic impact, particularly since player inactivity results in additional costs for medical treatment and influences team selection and player availability (Ekstrand, 2016). This in turn reduces team success both in the league and international competitions (Hägglund et al., 2013). As such, the implementation of prevention programs aims to reduce the negative implications of injury and consequently increase sporting success (Hägglund et al., 2013). Due to the importance of PTs in enhancing performance and minimizing injury, the objective of this study is to investigate the contemporary practices of PTs in professional soccer across two continents. This will help identify the methodologies used in the assessment of physical capabilities, monitoring and control of training, and injury prevention which will support the development of applied research and practice. In addition, the knowledge gained can provide a source of information for trainee PTs, with the data also being able to be used for current PTs for their practices.

## 2. Material and methods

The participants were asked to describe their practices and opinions, with the aim of providing an understanding of their knowledge and practices in professional soccer. The semi-structured interview script was submitted for review by five sports science experts. All reviewers were contacted *via* email and included the following: three reviewers with PhDs in sports science (two with experience in qualitative research design) and two reviewers presently operating as PTs. A pilot interview was also carried out with a PT. Minor modifications to the wording and organization of the survey were made to the interview questions following review and pilot testing to avoid conceptual ambiguities and ensure validity. Finally, the pilot test was incorporated into the analyses. The interview consisted of the following three sections: (1) evaluation of physical capacities; (2) training monitoring and control; and (3) injury prevention. The interview answers were all open and the PTs were able to share experiences and opinions on each of the questions. The study was carried out in accordance with the ethical standards of the Declaration of Helsinki and was approved by the scientific council and the ethics committee of the University of Coimbra (CE/FCDEF-UC/00692021).

Initially, 10 professional men's soccer PTs (Europe and South America) were contacted *via* email or telephone during the recruitment process. Of those 10 PTs, eight responded positively and agreed to participate in this research. Before conducting the interviews, all participants signed an informed consent agreeing to participate in the research and their interview was video-recorded. All interviews were conducted during January 2022 (the middle of the season for the European teams and the beginning of the season for the South American teams) and by the same researcher (JB). The PTs had a mean age (37.5 ± 5.8 years) with vast experience working in professional soccer (14.8 ± 6.4 years of experience). The participants had degrees in physical education (*n* = 7) and physiology (*n* = 1), and all had a master's degree or equivalent (*n* = 8), with some also possessing a doctorate (*n* = 3).

To be able to participate in this study, the PTs had to be working in a professional club at the time of data collection (first or second division and/or national team). Once eligibility and consent were obtained, participants engaged in a one-on-one semi-structured interview. Only entire interviews that were conducted from start to finish were used for analyses. The interview began with an explanation of the purpose, topics, estimated duration, and the anonymity of the information and its use (questionnaire available in Supplementary material). Each participant partook in an interview conducted *via* Zoom with the principal investigator, which lasted between 60 and 90 min (some interviews were prolonged since a few PTs provided lengthier responses to the questions). The structure remained similar for all interviews.

**Figure 1:**
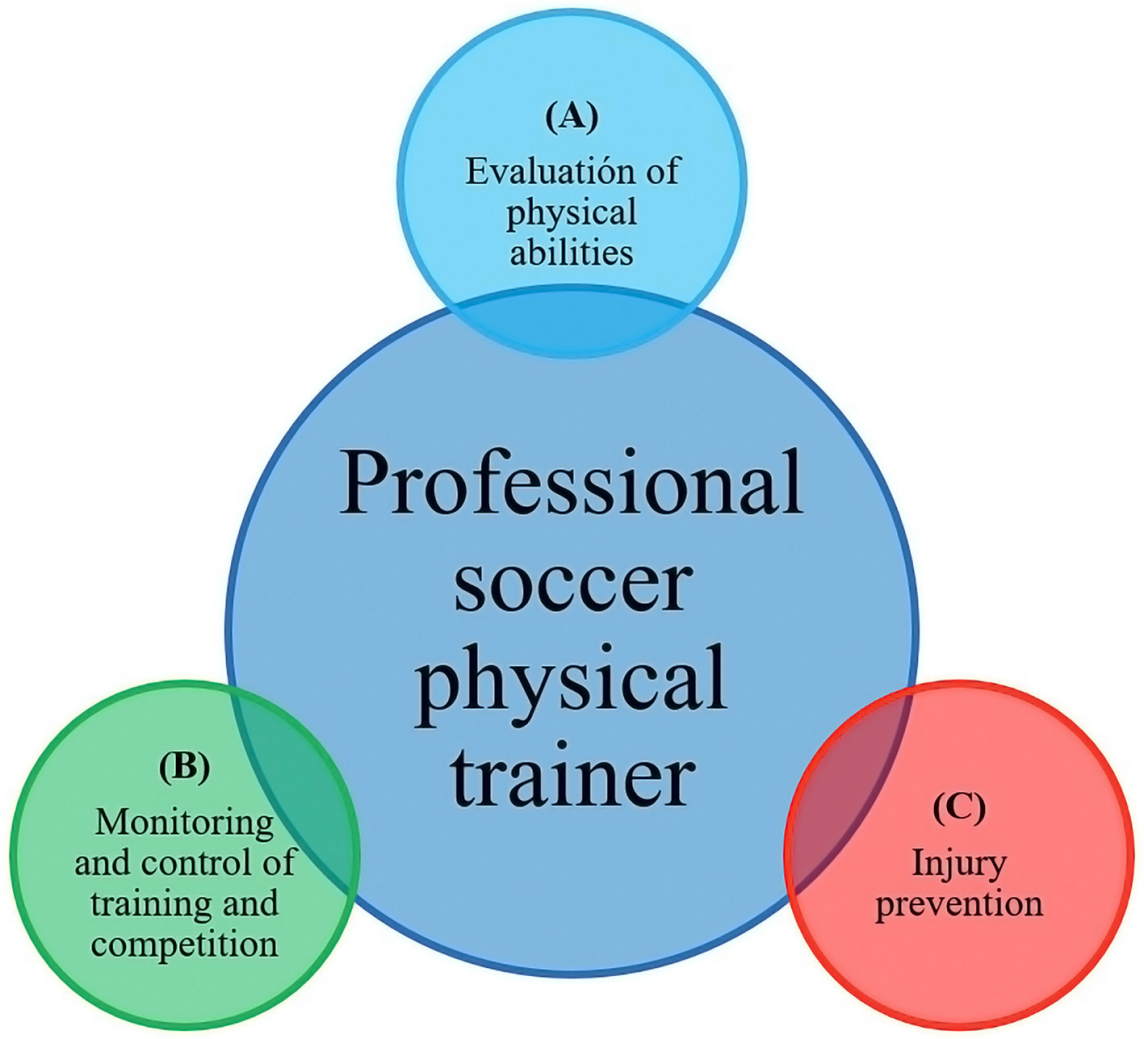
Dimensions that make up the work of the professional soccer physical trainer **(A)** Evaluation of physical abilities; **(B)** Monitoring and control of training and competition; and **(C)** Injury prevention.

To process the results, the qualitative content analysis technique was used to understand how the theory is applied in professional practice. All interviews were transcribed into a Word 2019 document (Microsoft Corporation, Redmond, WA) and coded using a deductive-inductive approach. The transcripts were read repeatedly to promote data familiarization and immersion in the underlying content (Creswell, 2007). The coding was based on a tree of nodes that was built to reflect the working models (Figure 1) (Côté et al., 2005; Fraser-Thomas et al., 2008; Henriksen et al., 2010, 2011; Davids et al., 2017) and mainly involved higher order issues. Similar to methodologies used previously, inductive coding expanded the node tree when new categories or ideas emerged; such categories and ideas primarily involved low-order topics and the content of topics (2010). The interviews and notes were then subjected to meaning condensation, in which participants' statements were condensed into precise formulations and a summary was written for each node. The QSR NVivo version 12 software was used to code the interview transcripts.

In this study, different techniques were used to establish reliability. The principal investigator received training in qualitative research methods described by several academic sources (Côté et al., 1993; Lincon, 1995; Creswell, 2007; Smith and Caddick, 2012). Member checks (i.e., the data were returned to participants to check for accuracy and resonance with their experiences) were undertaken to establish data credibility (Lincon, 1995). These checks occurred twice in the present study. The first took place during the information session that occurred at the end of each interview. At this point, participants were allowed to change any of their responses. The complete verbatim transcript of each interview was also sent to the participants for their final approval. At this point, the participants had another chance to clarify, add to, or delete any comments they had made during the interview. In addition, the intrapersonal reliability of the data was ensured by a panel of three experts in qualitative methodology who analyzed all units of meaning, themes, and categories.

## 3. Results

The eight participants worked for a club that competed in professional leagues on one of the two continents, namely, Europe (*n* = 4) and South America (*n* = 4). The main themes resulting from the qualitative content analysis are presented below.

### 3.1. Evaluation of physical abilities

#### 3.1.1. Relevance of carrying out evaluations for physical capacities

**Figure 2:**
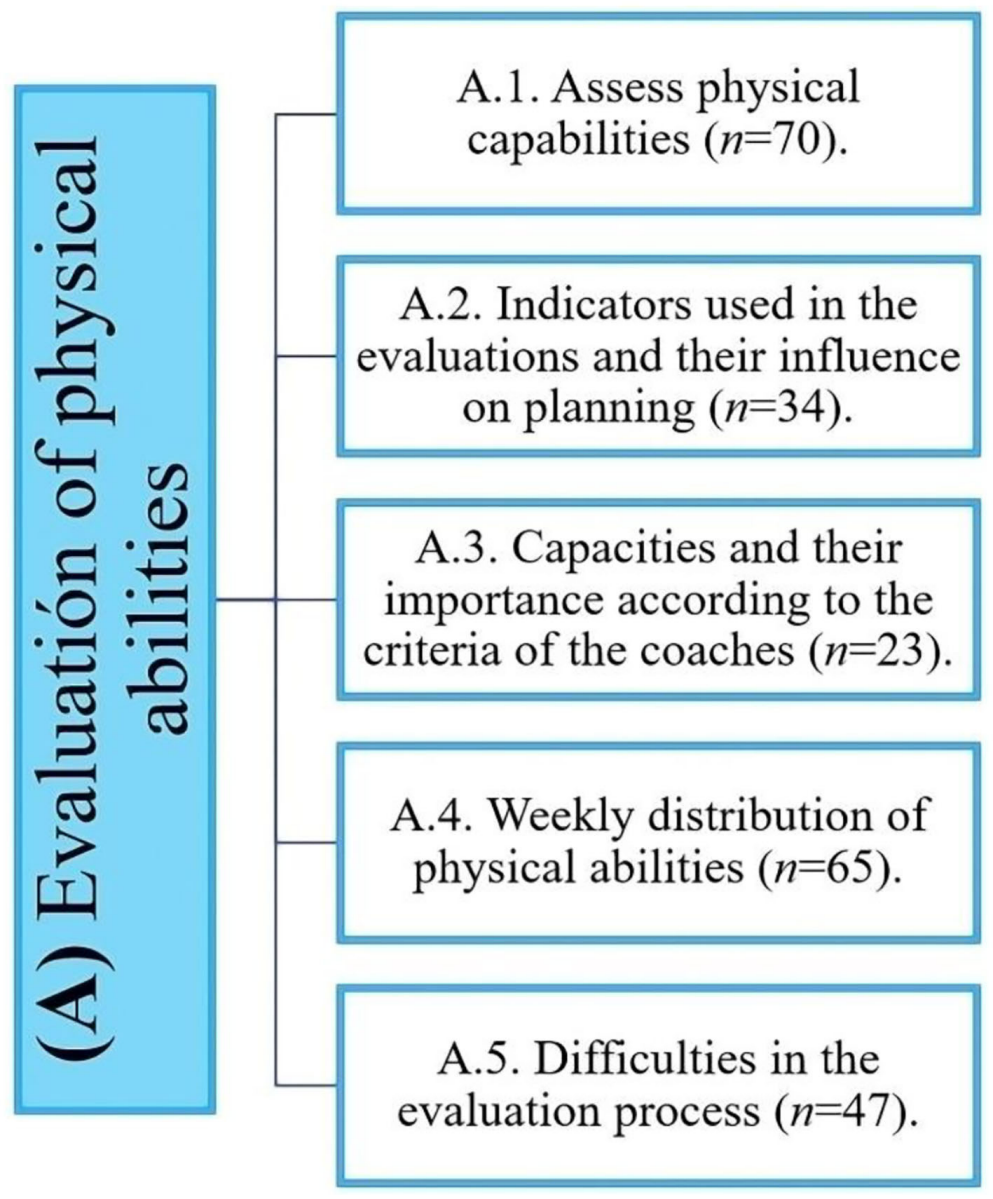
Distribution of the evidence obtained in the evaluation of physical abilities.

Regarding the “*Relevance of carrying out evaluations for physical capacities*”, we verified that it is a frequent process applied by PTs, who subjectively based on their beliefs, anecdotal experiences, and methodological preferences perform different evaluations that allow them to objectively measure and prescribe optimal training loads for their players (Figure 2).

Yes, always use tests to evaluate (...) with the objective above all to be able to prescribe training with more rigor, that was always the concern.

PT 6

As for the “*time of evaluations”*, PTs attempt to carry out evaluations at different times, such as the first at the beginning of the season (during the first or second week so as not to expose the players to maximum efforts), then again in the middle of the season (holidays), and lastly, at the end of the season, which in some cases may be conditioned by the decisions of the head coach's or the continuity of the players in the squad (contract termination and team changes).

(…) I do these diagnostic evaluations in the second week after a fitting so as not to subject the players to maximum effort after the holidays.

PT 2

In contrast, some PTs use partial evaluation strategies with sub-maximal evaluations or tests that do not generate great physical stress on the players. The aim of such work is to gradually adjust the prescription of training loads and limit the additional burden of training sessions, thus not retracting from the time when the head coach must implement their tactical strategy for the next match.

Another thing that I find interesting are the sub-maximal tests that I have already used many times, which are very easy to apply and are not so subject to this calendar variability, because they can be done in the initial part of the warm-up.

PT 7

Finally, “the physical tests that are used by PTs to determine the physical condition of the players” and that were considered *a priori* at the time of construction were anthropometry, agility, speed, aerobic capacity, anaerobic capacity, strength, and flexibility. The answers provided blood tests as an option *a posteriori*. The tests for anthropometry consist of the assessment of body mass, height, and the sum of six skinfolds. For agility, participants used the Illinois agility test and *t*-tests. The speed tests are linear and vary between 5, 10, 20, and 30 m. Aerobic capacity is estimated through evaluations of direct consumption and carried out in the laboratory with a maximum progressive test and in the field using the 30-15, the Yo-Yo test (both level 1 and 2 variations), or the 1,200 m test. Anaerobic capacity is determined by the 35-m Running-based Anaerobic Sprint Test (RAST), but there are others who do not consider it a necessary evaluation and do not perform it. Flexibility or range of motion is determined by the Functional Movement Screen (FMS). Force is determined using isokinetic tests to determine peak concentric and eccentric torque, as well as the Isometric Mid-Thigh Pull (IMTP). In addition, the neuromuscular performance of the lower limbs is determined through different jump evaluations such as the squat jump (SJ) and countermovement jump (CMJ). Finally, for the upper limbs, only one trainer indicated evaluating the maximum repetition test (RM) using the bench press.

(…) we performed some muscle function and neuromuscular performance tests such as the SJ and CMJ, isokinetic, then some dynamometry tests in an isometric regimen more related to hip abduction and abduction, and then a more general test that is related to very well with the performance that is the Isometric Mid-Thigh Pull (IMTP).

PT 8

In this same context, but using other reference indicators such as blood markers, PTs use these evaluations to determine muscle damage or stress level of the players before the start of the season or during periods of high match congestion, among other factors.

(...) I ask a laboratory technician to carry out blood tests to see how the players are when they arrive, to see if they are overstimulated, overtrained, stressed or not, based on being able to have this week of recognition from the players (...) I measure creatinine, ammonium, which are the stress hormones that tell you, look, this player is overtrained, you have to give him a break or, on the other hand, this player has not been stimulated for several weeks or a month.

PT 4

#### 3.1.2. Indicators used in the evaluations and their influence on planning

The use of data was diverse across participants and often depended on the preferences of each PT. In some cases, the use of information for planning ahead was to determine reference values at the beginning of the preseason process to inform subsequent training processes. This also included who should remain in the squad from the previous year or obtain a baseline for the new players. These data enabled comparisons later in the season to assess progress and highlight potential injury risk. Despite the intention of the PTs in the current study attempting to conduct end-of-season testing, there seems to be a consensus that it is challenging, given the high turnover of players and the accumulation of changes at the club.

(...) this helps us to have a diagnosis to know how they arrived, how the reinforcements are located, how they are located in the group.

PT 2

(...) the great objective is to try to determine if there is any type of muscular deficit in relation to one member to another.

PT 5

The information collected and analyzed allows decisions to be made that help to set goals or adjust training loads, mainly individually or by position, understanding that the demands in each positional role are different. Therefore, complementary work can be designed for the main training session, which is carried out according to their complexity before or after training, trying not to interfere with the work of the head coach, nor with the physical performance of the player.

Those who are above the average usually do maintenance work and those who are below average do complementary work logically associated with the metrics.

PT 6

#### 3.1.3. Capacities and their importance according to the criteria of the PT

Determining the importance of each physical component of the players is highly complex. If it was necessary to separate and analyze each one of the abilities according to their importance in the players' performance, two preferences were presented for the PTs: (1) strength is considered the most important capacity and it is the one that allows the achievement of greater sports performance. In addition, strength is a facilitating capacity for others such as speed and resistance; and (2) the importance of aerobic capacity is also highlighted due to the benefits it provides for rapid recovery after maximum effort, increased ability to repeat and tolerate these demands, and delayed the onset of fatigue and, in addition, its importance as a protective element to avoid injury.

Yes, strength (...) as a basic physical capacity, when we develop it, we know that we have greater contractile capacity, therefore, we are going to improve speed levels, we also have greater mitochondrial capacity, which is also going to help us with resistance.

PT 4

Another element that was considered relevant at this point is the mental capacity or cognitive and emotional wellbeing of the athletes. Specifically, it was deemed crucial to support the athletes' emotional wellbeing as this is the foundation for enhancing all other elements of the performance.

(...) I have an opinion regarding how the athlete should be in an emotional sense and my way of working is aimed first at having the athlete emotionally well, which for me is the basis of everything.

PT 2

#### 3.1.4. Difficulties in the evaluation process

The PTs refer to the challenges experienced across different phases of their professional development. Four key barriers to carry out evaluations were highlighted, with the first being player culture (i.e., buy-in and adherence). To overcome this challenge, the PTs conduct educational sessions on the importance of evaluating performance for athletic development to influence perceptions and subsequent engagement.

(...) the daily conversation, in the moments together of conversation when a trust begins to be produced and you begin to explain to him... he is going to be something in which you must have him at the maximum of the maxims of his will to carry them out, because if he did not have the will, it is preferable not to do anything because the data would not be valid.

PT 7

Another element pointed out as a factor that hinders the application of the evaluations is the club's own culture and standards. This can relate to the lack of economical allocation of resources allocated for assessing physical performance. Most PTs reported not to having adequate equipment to conduct their role and often the PTs themselves have to source equipment or facilities to implement evaluations.

(...) the resource, we did not have it in the club and I had to go out and look for it in clinics, sports centers that had a isokinetic device, but once it was achieved, when they gave me the values to evaluate each of the athletes, this It was escaping from the reality and possibilities of the club and from that point of view what I had to do was manage publicity to put in the stadium and lower the costs of the evaluation.

PT 4

Finally, there are also some problems at the operational level of different origins such as the structure of local and international competitions that can affect the planning, the fatigue of the players at a general level (physical and mental), and the change of opinion of the head coaches without prior notice and environmental factors.

(...) then there are other elements such as environmental ones, there must be optimal conditions to carry out the test, it is temperature, humidity, because they are things that influence the result.

PT 7

#### 3.1.5. Weekly distribution of physical abilities

Physical trainers consider that the structure of each microcycle should aim to improve all elements of performance, including technical, tactical, mental, emotional, and physical as part of a holistic model. PTs modify the structures of the drills that are undertaken by players by changing pitch/drill dimensions, number of players, durations, and goals depending on days within the weekly schedule (e.g., competition, day +1, day +2, day −4, day −3, day −2, day −1, and competition). Day +1 tends to be targeted toward promoting recovery (one day of active recovery and another day of passive recovery). Days −4 to −1 are days of optimization of elements of performance. In a microcycle with a mid-week match, more recovery days may be implemented within the schedule and fewer days may be allocated to performance optimization. Day +1 or +2 can be used for passive or active recovery, with PTs preferring active recovery on day +1 to speed up and facilitate the recovery process and passive recovery or rest on day +2. However, some head coaches prefer to carry out passive recovery on day +1 to promote psychological recovery after the competition and leave active recovery for day +2 being a training more intermittent and with a greater number of rest breaks that allow players to recover better before the following effort. In addition, the active recovery day can be used to balance the loads of the players who did not play or who played fewer minutes during the competition. At certain times, this can also be done after the competition on the field itself in a “cool down” format.

(...) the second day (+2) is of active recovery and a differentiation is made between the players who played the entire game and those who played < 45 min and did not play, must have a higher load.

PT 1

On day-4, trainers focus on physical conditioning training with a reduced number of players (e.g., 1 vs. 1 or 2 vs. 2), which involves accelerations, decelerations, duels for the ball, jumps, and changes of direction, interspersed with periods of reduced effort. The PTs declared that given that some players may not be 100% physically or emotionally recovered, it may be necessary to control and manipulate the intensity for certain players. This can involve different strategies, such as reducing the number of drills a player performs or providing the player with lighter duties within the session.

Strength work on day-4, there is a focus for tasks focused on accelerations and decelerations that cause explosive actions of short duration.

PT 8

Day-3, being the furthest day from the previous and subsequent competition, tends to involve larger structures and more players. This allows the main behaviors in the different phases to be developed more collectively (offensive and defensive transition) and moments of the game (offensive and defensive organization). It is intended that the players cover similar distances and reach maximum speeds consistent with those reached in competition. Data analysis can be carried out after training sessions to determine those that have not reached the established parameters. It is necessary to point out that for this type of control, it is necessary to have GPS devices that provide feedback in real time.

We call it the day of resistance, but at the base it is to create more specific contexts of everything that we want to work on in the game, therefore, resistance is not only aerobic resistance, it is a specific resistance, which later moments of the game will require us for the different positions (...) we try to achieve what is known as speed running that we achieve during the competition.

PT 5

Day-2 is oriented to higher speed actions, which can be achieved in a specific way, but with low complexity in decision-making.

Finishing exercises with pursuit or more analytical linear running exercises of 30 m or more, with long recovery periods and reduced execution periods, are usually used.

On a more speed basis, not only in analytical-type actions, but also with more specific finishing actions, arrivals to the area and faster actions with a short duration (time) but with enough recovery time between the different actions.

PT 5

Day-1 is oriented to active recovery or match activation. This day together with the speed day is also recognized as tapering and less of a physical impetus. The day before the competition is also focused on completing match preparation, with tasks focused on reaction speed and covering more detailed elements of the competition from a psychological, technical, and tactical preparation.

The last day, which is a little more focused on the socio-affective part, the work of some tactical concepts of stopped ball and in the activation, we make games that are more oriented in the union of the group and the creation of bonds between the players, to be able to go all united to compete with the rival on duty.

PT 2

During the two match microcycles, recovery becomes much more of a focus when compared with one game week. The main emphasis of the week is to optimize performance, but it was also a key priority in managing physical elements that help maintain performance such as reducing fatigue, overreaching, and injuries. For this, PTs use different strategies that range from reducing the duration of exercises and training sessions to combining days off with different stimuli such as strength with resistance or speed with activation, or strategies focused on the methodology of the head coach.

(...) at certain times you intend to recover, but it may be more important to adjust certain situations or certain technical-tactical behaviors than to have the players 100% recovered (...) the challenge for the head coach is planning and creating training, in order to obtain the greatest possible fruit, with the lowest cost.

PT 7

### 3.2. Monitoring and control of training and competition

#### 3.2.1. Use of monitoring instruments

**Figure 3:**
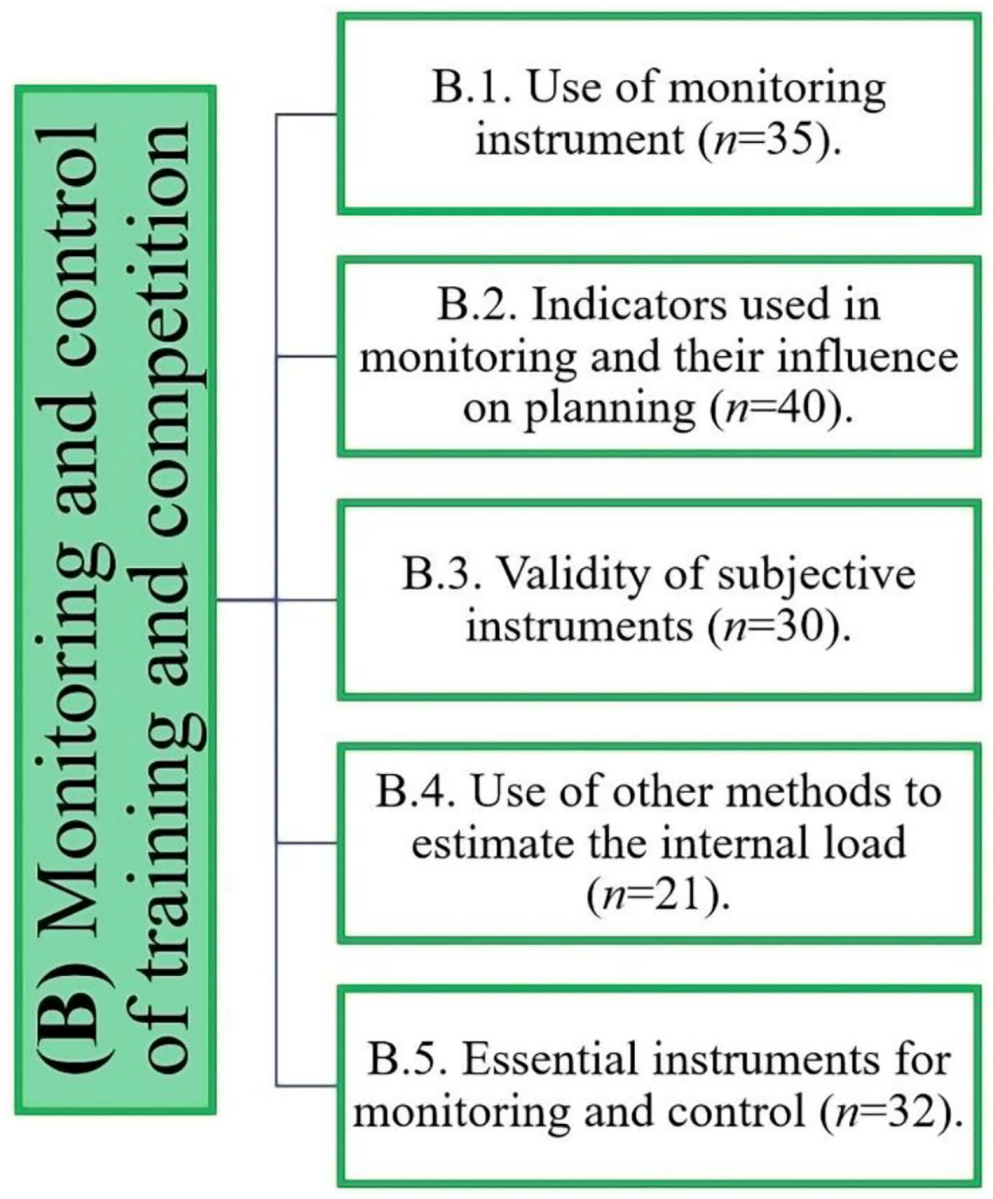
Distribution of the evidence obtained on the monitoring and control of training and competition.

Monitoring and control of training have become fundamental factors in soccer for maintaining high performance in matches and managing players' workloads (Figure 3). There was a revelation that the practitioners used monitoring methods that focused primarily on three factors, including subjectively assessing the player's wellbeing prior to each training session, monitoring GPS activity throughout the session for objectivity, and analyzing data post-session.

Before the session, “*the use of wellbeing questionnaires”* is implemented to minimize fatigue and injury risk and maximize a player's physical and emotional wellbeing.

(...) we use the Wellness questionnaire, which allows us to know what conditions the athlete is in to carry out the training session.

PT 1

During the session, “external load control” can be monitored in real time or after the training session on the premise that the club possesses “live” feedback systems. A consensus existed in the current study that practitioners used GPS devices, with the training session controlled by the quantification of specific parameters (i.e., total distance, accelerations, decelerations, maximum speed, and distances at high and very high intensity).

GPS for external load, (...) I chose to take only some indicators (...) first the total distance, then (...) ranges of higher speeds that would be maximum and sub-maximum and then (...) accelerations and great accelerations and also finally the maximum speed reached.

PT 7

“Internal load control” was determined by the subjective perception of effort (CR10 scale). For this process, the PTs recommend individualized protocols, which were applied 30 min after training so as not to reduce the influence of scoring associated with collecting data immediately after a session. Players were educated and familiarized with scales in advance of use to ensure accurate interpretations and valid data. The value of using heart rate as an important objective monitoring tool was recognized among the practitioners, but this metric is often omitted given the discomfort associated with wearing these devices on the front. Some PTs used blood markers such as CK to determine muscle damage, but it is generally used at times when more than one match per microcycle is completed. Blood lactate was not used for monitoring due to the complex application and the information deemed not necessary for the planning process.

(...) post-training we applied the subjective effort perception scale, and there we had important data on how the player felt the training load.

PT 2

#### 3.2.2. Indicators used in monitoring and their influence on planning

The information collected from the training session is used to estimate load and determine whether the weekly load is within optimal parameters to promote adaptation and reduce overreaching. At the statistical level, the information is used to make comparisons between players and microcycles or between sessions and competitions and then to estimate metrics such as the Z-score to assess where the player is in relation to the team average and specific positional roles.

(...) the metrics are organized according to the competition, (...) this allows us to evaluate more from the performance point of view.

PT 6

At a collective level, monitoring allows a more general view of the total load of the session, competition, or microcycle, but the major adjustments are made to individuals. This may be attributed to the understanding that the specificity of playing positions requires stimuli and demands that vary. In this sense, the information is analyzed to adjust the loads when the expected values are not reached or exceeded.

(...) the individual data allowed us to know which athlete we had to raise or lower the load.

PT 2

#### 3.2.3. Validity of subjective instruments

Subjective evaluation instruments are recognized, used, and validated by all PTs on a daily basis; however, they make some recommendations in their application. For instance, the application of the questionnaire must be applied to a certain degree of privacy so as to reduce bias. The RPE should be taken and recorded approximately 30 min after the end of the session or competition. The subjective monitoring questionnaires should also be shortened where possible to increase engagement. The results that appear substantially different from the group average are questioned and addressed with the player in an effort to understand the nature of the response, given the complexity of collecting subjective data.

The evaluation of the effort perception scale or any other tool that gives me the proximity to what is happening to the athlete is going to be crucial for me not to make a mistake and give the head coach a tired athlete at the time of the competition.

PT 4

#### 3.2.4. Use of other internal load indicators

In general, the trainers value and used heart rate monitors. However, all the trainers made the same comment related to the lack of comfort of the monitors/bands, interfering with player buy-in. CK was the most used blood markers to determine muscle damage, but it is generally used when there is more than one competition per week to try to help in the recovery process without creating overloads in the players and to reduce the invasion of drawing blood weekly.

Yes, we already use the cardio frequency meter and we stopped using it because of the discomfort expressed by the players.

PT 5

We use the CK indicator as an indirect measure of muscle damage.

PT 8

Other tools that PTs have used to monitor players and determine the degree of recovery range are sleep analysis with an electroencephalogram (EEG), hydration levels with a refractometer, and estimated neuromuscular capacity with a jumping platform.

(...) strength is also a good indicator to determine fatigue and that is why we use jump ability as a method to evaluate 48 h after the game.

PT 3

In this same sense, an indicator that is not used and to which PTs do not give importance is the measurement of lactate, which in their opinion is not practical to be applied in the normal training process.

Lactate for me is not useful and impractical and I don't use it.

PT 7

#### 3.2.5. Fundamental elements for monitoring

The PTs highlighted that GPS devices are crucial to obtain this information ideally in real time to make decisions that allow them to quickly adjust loads.

(...) the GPS would be to control the external load and have this possibility of controlling them at the moment.

PT 2

At the internal load level, the use of the different subjective perception questionnaires is valued and validated and was applied before and after training sessions.

(...) the Borg Scale, Wellness Questionnaire are simple things that I am already familiar with and use and have worked for me, because I believe that sometimes less is more.

PT 2

In this same sense, the heart rate appears as a valid and objective indicator of effort at the internal level, for which its value is recognized and its use is recommended, as well as CK analysis to determine muscle damage.

(...) heart rate, because it is an objective measure and is more valid, can be influenced by various factors, but during training, it is logically a valid indicator.

PT 7

Other elements that PTs consider important for this process are the creation of multidisciplinary departments that allow players to be monitored in a more comprehensive way. They revealed that this should include nutritionists, physiologists, doctors, and coaching staff to thoroughly analyze the preparation strategies, training processes, and recovery regimes.

(...) first a good monitoring center, (...) with a physiologist, physical trainer, a monitoring manager generating a competitive department... internal, debatable where all the variables are analyzed very thoroughly.

PT 3

### 3.3. Injury prevention

#### 3.3.1. Main causes of injury

**Figure 4:**
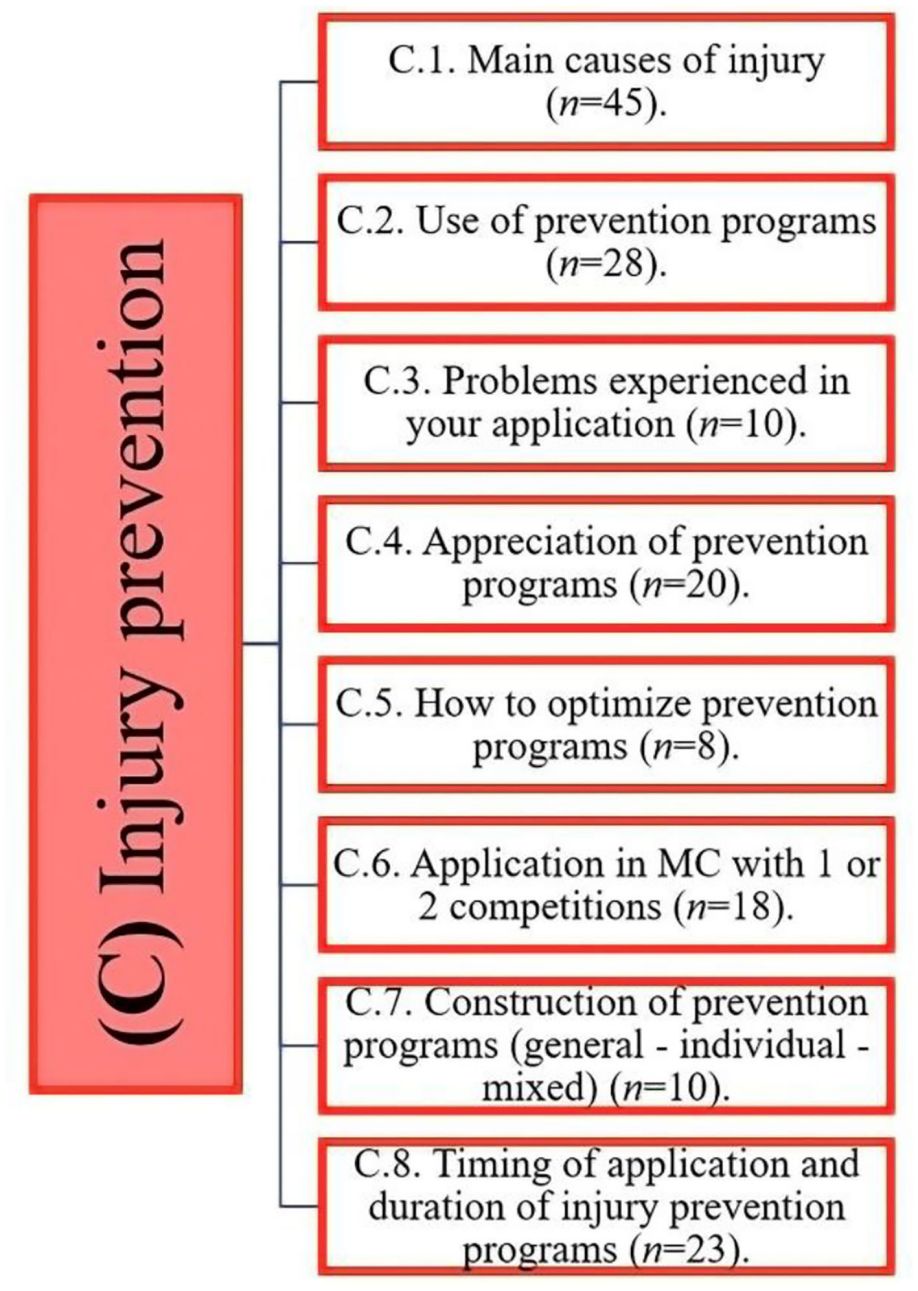
Distribution of the evidence obtained on injury prevention programs.

The PTs highlighted three main elements that are considered most important to minimize injury risk, including, previous injury, age, and fatigue. Other less commonly highlighted factors were poor recovery, match congestion, contextual variables, and psychological factors (Figure 4).

If an athlete has already had an injury, the probability of suffering another injury is very high, therefore, the care with that player must be doubled.

PT 6

Physical trainers also consider other elements as risk factors for injury, such as low levels of oxygen consumption, high levels of body fat and low levels of muscle mass, returning to competition prematurely, methodological elements (absence of preventive work), dangerous training processes, poor diet and rest habits, muscle deficits, overload, and underload.

Players with a low level of oxygen consumption are more likely to suffer an injury than a player with a good oxygen consumption.

PT 1

(...) deficits at the muscular level, it also seems to me something very relevant or something to which we give a lot of relevance... if a player has a lot of difference or significant differences between the lower limbs, it can be a sign that he will suffer an injury in a period next.

PT 5

#### 3.3.2. Use of prevention programs

Injury prevention programs used by PTs focus on those considered multicomponent, since they consider different elements, such as central strengthening (CORE), eccentric exercises, proprioceptive exercises, running technique work, speed, total-body resistance exercise (TRX), and FMS. However, some coaches prefer not to refer to these programs as preventive, but rather as optimization programs. This appears to be linked to their focus on improving the ability of the players, which will secondarily allow them to be better conditioned to the demands of the game.

(...) we have multicomponent injury prevention programs, that is, that use those exercises that are described in the literature, such as the Nordic exercises, Copenhagen abduction, balance exercises, some FIFA 11+ exercises (...), using those exercises that have already been validated and that have some efficacy in preventing injuries and we do it that way.

PT 8

The PTs refer to other elements that they consider fundamental within the prevention process that are due to the training methodology used. The alternation of loads is completed from the day of active recovery (+2 or +1) and up to the activation session (−1).

When training in a contextualized way, the muscle creates adaptations in specificity, that is, the muscle fiber adapts to the effort or stimulus that is required. This is essential.

PT 1

#### 3.3.3. Problems experienced by the physical trainer in the application of preventive programs

The participation of the players in a professional context does not seem to be subject to great difficulties since, due to contractual elements, the players must carry out the activities indicated by the technical staff who leads the sports project. For this reason, participation in prevention programs is high and absences are relatively low and motivated by specific reasons.

In contrast, they highlight the importance of accurately instructing players during their training process (e.g., young players) on the importance of these preventive works, so that it becomes a self-care habit that lasts throughout their career.

I haven't had any problems, I think that the different teams have been working well for a long time and the players, despite changing clubs, understand that before training they have to do some exercises that are important for their health.

PT 2

However, the problems that some PTs have experienced are punctual, such as (1) assignment of the players in different work groups (headlines—reserves), which generates poor status in some reserves; (2) some young players who did not have good professional training related to injury prevention issues and, therefore, are not used to it; (3) minimal human resources to control and guide tasks; (4) refusal of some specific exercises; and (5) the sports context (team or selection).

(...) there are specific exercises with which we later have more difficulties, specifically the Nordic hamstring exercises.

PT 8

#### 3.3.4. Appreciation of prevention programs

The assessment and acceptance of injury prevention programs by head coaches and the medical staff according to PTs are quite high. In the case of the head coach, having all the players available and in optimal conditions is essential to achieve the sporting objectives. However, PTs recognize that depending on the professional training and the head coach's own experience as a player, sometimes they generate some difficulty, so it is essential to justify and explain the relevance of this process. In contrast, the medical departments are key in this process, since their contribution, experience, and knowledge of the players who have been with the club for a longer time help to better optimize the process. In this sense, there is agreement that the implementation of prevention programs should be designed by a multidisciplinary team considering the maximum amount of information from athletes for greater optimization and effectiveness.

(...) with the medical team this is done in direct collaboration and many times we adjust the training programs because they are carried out on a daily basis (...) we adjust in relation to the complaints or problems that there are from the players in the medical department, to clinical level… for this reason there is a 100% interaction with the medical department.

PT 8

#### 3.3.5. How to optimize prevention programs

There is agreement that injury prevention programs can be optimized to obtain the maximum benefit from their application, whether from a prevention-oriented perspective or the improvement of the athlete's physical capacity. PTs use various strategies such as (i) ensuring the work is soccer-specific and simulates the movement patterns performed during a match; (ii) design programs that are carefully implemented to determine a balance with workload, fatigue, rest, and nutrition; (iii) consider all risk factors, both internal and external; (iv) create harmonious, dynamic, and engaging programs that facilitate their application; (v) elements of the medical department such as kinesiologists or physiotherapists should be incorporated to have a more proactive role in injury prevention; (vi) train and educate academy players on the importance of prevention programs to adhere to such protocols as they progress to the professional level; and (vii) individualize the prevention programs for each player, attending to their needs and abilities.

(...) Preventive work must be related to the main tasks (specific field work), since preventive tasks are complementary to the main tasks and must have a certain link with the type of muscle contraction that is applied.

PT 1

#### 3.3.6. Application in microcycle with 1 or 2 competitions

The application of prevention programs is mainly based on whether teams compete in a one- or two-game microcycle, with PTs adjusting loads accordingly. Thus, a maintenance injury prevention program is carried out on one match microcycles, but not on a two-game weekly schedule to avoid negatively influencing player wellbeing.

By having a standard microcycle with competition from Sunday to Sunday, the application of prevention programs is normal, considering the different elements that were mentioned above depending on the day of the week.

PT 1

To reduce load, alterations are made to compensate for the additional game in a competitive microcycle with two matches per week. These measures are used in order to promote physical recovery for the next competitive encounter, allowing the player to focus on the more technical and tactical aspects of the next game.

There is a substantial reduction in the volume of the load of these prevention programs in the gym. Many times they stop carrying out these programs in congested periods of the sports or competitive calendar... this manifests itself with a reduction or a complete absence on certain days of these programs.

PT 8

#### 3.3.7. Construction of injury prevention programs

Physical trainers consider two major aspects when constructing prevention programs. The first relates to elements of the PTs' own learning and their scientific knowledge on this subject, which is used in a broader and more general context. The second adds individual factors to these elements, such as injury history, and anthropometric or physical evaluation results to determine imbalances that may affect the player's health. Consequently, the trainers, when considering both elements, consider that the construction of their prevention programs is of a mixed nature, since they articulate both elements when constructing these programs.

We did general days in which the work for the entire team related to strength and they also had individual work guidelines for each one, based on the body composition evaluations, the FMS evaluation, the injury questionnaire and your previous injuries, to strengthen your irrigation areas.

PT 2

#### 3.3.8. Timing of application and duration of injury prevention programs

The moment of application of the prevention programs is mainly oriented to be carried out prior to the main training session, mainly when the work is oriented toward the lower limbs to activate and prepare the neuromuscular system for an intense session. Another factor is related to the willingness of players to work before the field session and not after. However, there is also the possibility that these programs are carried out after the main training session when double shifts are carried out or when the muscles need to be worked to correspond to the upper limbs.

(...) the player in general prefers to arrive earlier and do this work before, instead of doing more work after the training session.

PT 5

The estimated duration of the prevention work can fluctuate between 10 and 35 min, depending on the day of the week in which it is being carried out. Another factor that influences the duration of the prevention program relates to the player who is carrying out the session and the context involved with whether the player required greater work or whether recovery is a higher priority.

It depends on the type of work it is, it can last 10, 15, or 20 min depending on the work that is done.

PT 6

## 4. Discussion

**Figure 5:**
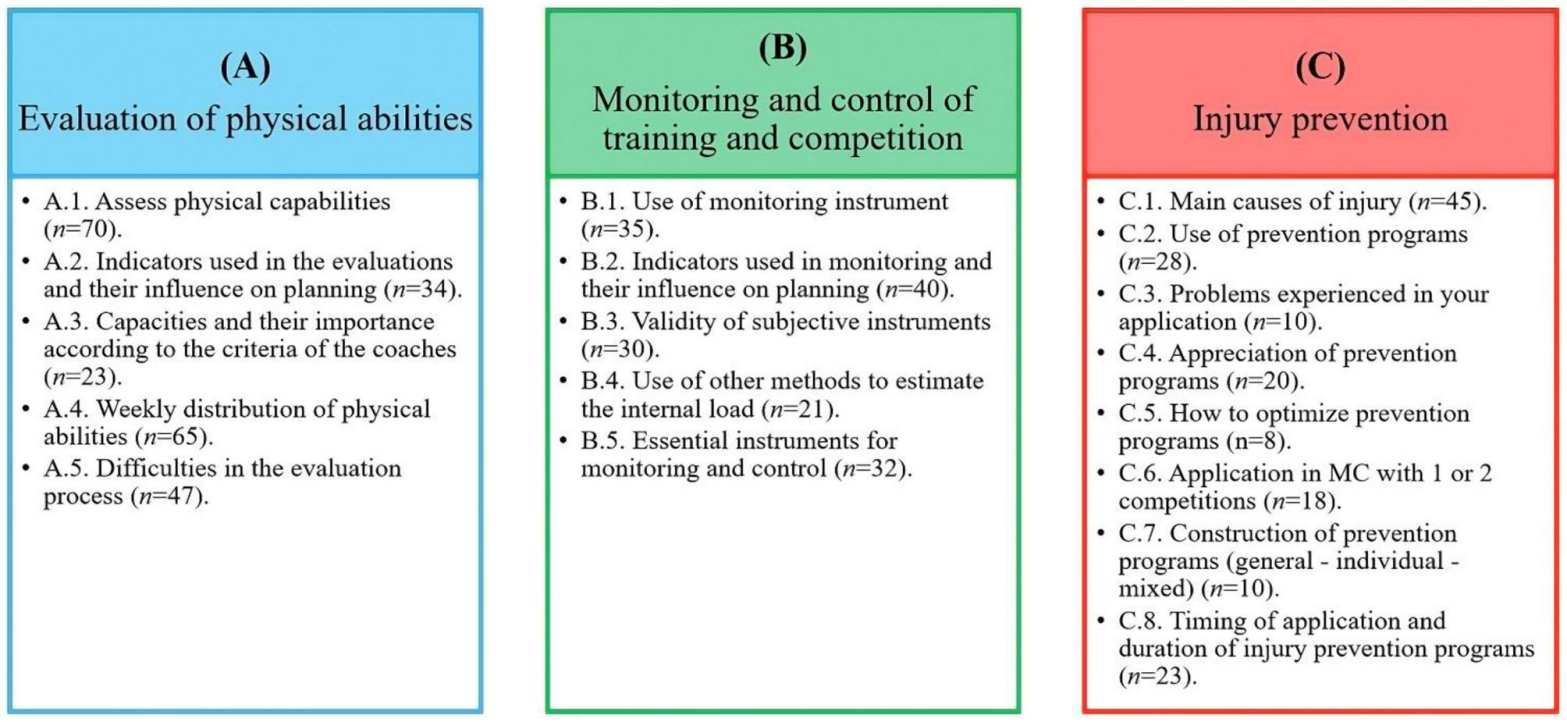
Summary of the findings found in the three areas studied **(A)** Evaluation of physical abilities; **(B)** Monitoring and control of training and competition; and **(C)** Injury prevention.

The aim of this study was to investigate the contemporary practices of professional soccer PTs across two continents. This study provides an understanding of the contemporary practices of PTs from two continents and different leagues. The PTs presented an extensive period of experience at a professional level and were educated to a high academic level. This provides an understanding of how these PTs implement the theory acquired in their training process into practice and how they adjust according to their experience in the professional context in three areas (Figure 5).

### 4.1. Anthropometric evaluation

The anthropometric and body composition assessments are considered an important indicator of fitness for PTs. This was considered an important aspect of physical fitness for soccer by the current participants, since the superfluous adipose tissue can act as dead weight in activities in which body mass is repeatedly raised against gravity when running or jumping during play (Reilly, 1994). In addition, possessing favorable body composition (high muscle mass and low-fat indices) has been considered an important selective factor to achieving success in sports (Rienzi et al., 2000). However, caution is advised when interpreting such claims, as each successful athlete may possess different physical characteristics (Rienzi et al., 2000). Therefore, each PT assigned less importance to this matter, assuming that other physical performance parameters were adequate. Practitioners used skinfold assessments for assessing body composition. Skinfolds, although less reputable than other more sophisticated methods (e.g., DEXA), have been advocated for their simplicity and day-to-day consistency (Kasper et al., 2021). Minimal information was provided on how the practitioners might use the data obtained to determine subsequent nutritional and training protocols. Therefore, more studies appear to be warranted to determine how skinfold data are used to inform practice and delve into the specific role of the PT in relation to skinfold practices to enhance the understanding of this key undertaking within the applied setting.

### 4.2. Physical evaluation

To cope with the physical demands of soccer, players must be able to adapt to unpredictable changes and unorthodox movement patterns (Drust et al., 2007), which increase energy expenditure over anticipated locomotion patterns (Reilly and Bowen, 1984). Such movements can include acceleration and braking, forward, backward, or sideways actions, and cutting and side-stepping maneuvers to evade the opponent's defensive marking (Drust et al., 2007). The use of physical fitness tests helps to examine the abilities of soccer players (strength, power, speed, agility, and aerobic and anaerobic capacity) and training programs to be better targeted. This is even more crucial given the complex nature of soccer (Svensson and Drust, 2005). The evaluations can be applied both in the laboratory context, which is usually more expensive and sensitive, and in the field, which is carried out by the trainers themselves at a lower cost (Drust et al., 2007). The evaluations of the physical capacities of the players were highly supported and used by the PTs to provide a more comprehensive depiction of player fatigue and whether recovery should be prioritized akin to previous research (Viru and Viru, 2001). This careful monitoring allows practitioners to demonstrate the evolution of the training process and information to make more accurate decisions about training prescription (Svensson and Drust, 2005). Such evaluations provide a logical framework for the use of performance tests that better understand the physiological demands of soccer (Drust et al., 2007). The PTs' interviews revealed that there is also a classification by positions for a more accurate and specific analysis of the demands of competition, as suggested within the literature (Rienzi et al., 2000; Barrera et al., 2021). In this sense, and despite the difficulties that the participants may experience during the evaluation process (instrument, logistics, cultural, among others), they continue to be used to try to understand the player's performance capabilities. The practitioners tended to favor field-based tests that are more sport-specific, given their acyclic nature that is not amenable to time-series analysis and is incompatible with traditional exercise study models in laboratory conditions (Svensson and Drust, 2005).

A key laboratory test that was commonly used among practitioners, provided that the necessary resources were available for this purpose, was isokinetic dynamometry evaluations of the lower limbs to determine peak torque and muscle imbalances (strength), specifically around the main knee extensor and flexor muscles (quadriceps and hamstrings) (Duarte et al., 2018a,b; Rosa et al., 2022). Evaluation of adductors and abductors with a dynamometer (Smart Groin Trainer) to identify imbalances or muscle weakness and monitor the progression of optimization and rehabilitations (Rosa et al., 2022) and neuromuscular capacity assessments *via* CMJs and SJs were also performed (Jovanovic et al., 2011; Loturco et al., 2018; Boraczynski et al., 2020; Rosa et al., 2022). The field-based tests carried out focused on speed over numerous distances (5, 10, 20, or 30 m), agility (Illinois T), and aerobic capacity (Yo-Yo test), which have been widely used in the scientific literature (Little and Williams, 2005; Mirkov et al., 2008; Kaplan et al., 2009; Jovanovic et al., 2011). This shows consistency between the practices employed and the scientific evidence base. When asked about the importance of specific physical abilities over others, the vast majority highlighted strength as the key fitness component, since matches incorporate explosive efforts such as sprints, jumps, tackles, kicks, changes of pace, and duels for ball possession (Cometti et al., 2001) to ensure successful performance in competition (Papaevangelou et al., 2012). Therefore, the ability to generate explosive muscular force in as short a time frame as possible is an important determinant of performance (Thorlund et al., 2009). However, some PTs also highlighted the relevance of aerobic capacity as an important element to ensure players can tolerate fatigue and recover more quickly (López-Revelo and Cuaspa-Burgos, 2018), as well as its benefits as a moderating factor of injuries (Malone et al., 2017a; Windt et al., 2017). These revelations are plausible, as both abilities are considered important determinants of soccer performance, as they are key characteristics of physical ability and the main regulators of important soccer-specific tasks (Hoff and Helgerud, 2004). The PTs interviewed revealed the importance of evaluating physical abilities from both a performance and injury prevention and rehabilitation perspective to support decision-making in the return to competition. It is recommended that the optimization process focuses on strength and aerobic capacities that, for the PTs, appears to be more predominantly targeted toward achieving sporting success.

### 4.3. Monitoring and control of training and competition

In the world of sports, the main objective of athletes and coaches is to achieve success in competition. However, by increasing the frequency, duration, or intensity of training, they risk creating excessive fatigue that can lead to functional impairment, described as “overtraining syndrome”, “staleness”, or “burnout” (Hooper et al., 1995). Therefore, it is essential that PT permanently monitors and controls the training process, since this process helps establish a balance between performance and recovery (Gaudino et al., 2015a; Moalla et al., 2016). This method also allows control over weekly workloads, which enhances physical conditioning and prevents injuries or illnesses (Jaspers et al., 2017). However, achieving these goals requires careful planning and manipulation of the training load over the course of a competition schedule (Gabbett, 2016).

All PTs noted that GPS devices are key to the monitoring process, consistent with the literature and reported use in Europe, the United States, and Australia (Akenhead and Nassis, 2016). Reasons given for the use of such units were the attainment of valid data (Reinhardt et al., 2019), data are reliable and relevant to track external load in professional soccer, both collectively and individually (Rave et al., 2020), and the device allows the measurement of positions, speeds, and movement patterns of the players (Cummins et al., 2013). Differentiation of the specific loads is recommended according to the individual demands of the game or position (Barrera et al., 2021); the evidence is supported and shared by PTs.

It was also highlighted that the practitioners use subjective tools, which allows them to understand how the player is reacting to certain external loads and also obtain information on the training performance that can be expected from individual players during a training session (Malone et al., 2018), during different days and weeks, and also at different times of the season (Nobari et al., 2021). For this process, the PTs mainly use two subjective perception questionnaires. The first is the wellness questionnaire that provides information about the athlete before the training session and competition and the level of fatigue experienced (Cullen et al., 2021). It is important to note that the PTs recommend adopting or eliminating some questions according to their personal criteria (subjectively), so as to lessen the time burden of irrelevant questions. Second, the Subjective Perception Scale (session-RPE) proposed by Foster et al. (Foster et al., 1995, 1996, 2001; Foster, 1998) is used post-training and competition to quantify the load of the session at an acute level. This is considered a good indicator of the global internal load of soccer training (Impellizzeri et al., 2004; Casamichana et al., 2013; Fanchini et al., 2017), using the category proportion scale (CR10 scale) (Borg et al., 1987) for its duration. It was also revealed that educating players about the importance of this information for the process of planning and executing training is key to ensuring engagement. For this to occur, the PTs indicated that this information is not used to determine starting, substitutions, or non-squad players in the subsequent match, but instead the veracity of the information is important for an adequate optimization and care process.

The PTs stated that they do not use the acute-chronic load relationship for training control. A practice that we believe should be incorporated, since it has been shown that high chronic workloads can be useful as a control parameter for injury prevention when adequately achieved in professional athletes of all sports, such as rugby players (Hulin et al., 2016), Gaelic footballers (Malone et al., 2017b), and soccer players (Malone et al., 2017a), which can be a fundamental tool to incorporate into practice. The PTs also considered the use of heart rate important to estimate the internal load in a more precise and objective way and to plan training sessions, in addition to being an individual indicator of the assimilation of accumulated workloads (Naranjo et al., 2015). However, it was reported that the players feel discomfort when using the elastic bands on their chest, since it is necessary to adjust them constantly, in turn, making their application difficult throughout the season. This perhaps offers an avenue for future research with technology companies adjusting the products to ensure player comfort.

Some PTs who possessed the resources used CK as an indirect blood marker of muscle damage and overload in soccer players (Lazarim et al., 2009b; Thorpe and Sunderland, 2012), in conjunction with other measures (e.g., CR10 and wellness). This marker was typically used within two-game microcycles. However, contrary to anecdotal belief, PTs indicate that they do not use blood lactate due to the perception that this marker is impractical, and the information provided is not considered relevant for daily training planning and prescription. Coutts et al. (2009) pointed out that lactate alone cannot accurately explain the overall intensity of soccer exercises, but that the subjective perception of the players' effort, which is a simple, cheap, and easy-to-use tool, should be used as a more accurate indication. Finally, it should be mentioned that all this information is considered in the training planning process, both individually and collectively. It was also shown that wellbeing values far from the mean, as well as workload peaks, will force modifications within the training process, individually or collectively, since players respond differently to the same training stimuli (Mäestu et al., 2005).

In general, it must be understood that the use of technological tools for the estimation of the internal and external load has become essential in high performance. However, in the absence of economic resources, other options such as subjective questionnaires can be used to replace the indicators of internal load or recovery. In contrast, if the resources exist and it is intended to provide greater comfort to the athlete, there may be other options for monitoring the external load (i.e., camera-based systems). It is important that monitoring and control must be present in the training process, since they directly impact the planning of each session, whether microcycle or mesocycle, if the intention is to reach the maximum sporting level with the lowest possible risk of injury to players.

### 4.4. Injury prevention

Injury etiology models have evolved over the last few decades, highlighting a number of factors that contribute to the causal mechanisms of sports injuries (Windt and Gabbett, 2017). Three factors are highlighted: (1) internal modifiable and nonmodifiable risk factors (e.g., age and neuromuscular control); (2) exposure to external risk factors (e.g., playing surface and equipment); and (3) inciting events, in which biomechanical breakdown and injury occur (Meeuwisse, 1994; Meeuwisse et al., 2007). In this context, the PTs indicated intrinsic risk factors, i.e., the history of injuries and the age of the players, and extrinsic risk factors, i.e., the fatigue caused by training and the high congestion of matches (Dvorak et al., 2016). However, it should be noted that the PTs highlighted the complexity of the injuries and their unpredictability, which makes them a complex problem to predict and address.

As a product of the high complexity of injuries and how important it is to maintain low injury rates for sporting success, as well as for the economic sustainability of the club (Ekstrand, 2016), the PTs pointed out that they use multicomponent programs, which incorporate different exercises for different types of injuries that a player can suffer. It was also stated that these programs build on the basis of scientific evidence focusing on two topics: (1) main injuries that occur in soccer (e.g., groin injuries, joint injuries, and muscle injuries) (Chomiak et al., 2016) and (2) greater prevention benefits in these injuries, such as the Copenhagen Adduction exercise programs (Harøy et al., 2019), Nordic hamstring exercises (Al Attar et al., 2017a), reverse Nordic hamstring exercises (Alonso-Fernandez et al., 2018), knee-specific training modules (Krutsch et al., 2020), as well as the training methodology focused mainly on meeting the specific needs of the sports (e.g., accelerations, decelerations, distance covered at high intensity, and sprints performed, among others). The PTs indicated that the participation of the players in these programs is high, which eliminates the problems or difficulties that may arise due to low adherence (Engebretsen et al., 2008). These programs are carried out mainly before the training session, despite the fact that evidence has shown greater benefits against new injuries when applying for prevention programs before and after the training session (Al Attar et al., 2017b) and have a duration between 15 and 30 min. However, the duration times according to the literature do not influence the positive results of application in preventative programs for injuries suffered in the lower limbs, the type, severity, or mechanism of the injury (Rahlf and Zech, 2020). The configuration of injury prevention programs can vary depending on the day of their application within a normal microcycle (one competition) as well as in a microcycle with two competitions per week. Programs can often be overlooked during the fixture-congested periods of matches depending on the PT, and these variations have been shown to affect the effectiveness of programs (Van Beijsterveldt et al., 2013). These programs are designed in a specific way depending on the needs of each player and the demands of the different structures (Alcalá et al., 2020), which has shown favorable results against muscle injuries and days of absence without recurrence (Melegati et al., 2013). Therefore, it is essential to consider the flexibility of the planning and delivery of these programs in order to achieve the benefits of these injury prevention programs.

The PTs believed that both head coaches and members of the medical team value the work done when using these prevention programs and highlight the importance of having a more interdisciplinary approach that involves different members in the optimizing process to reduce the risks related to the sports discipline given its high complexity, as suggested by the scientific evidence (Dvorak et al., 2016). Finally, the interviewed PTs believe that prevention programs can address the more specific demands of soccer, as proposed by Moras (Moras, 2000; Seirul-lo, 2017), with the degrees of approximation of the tasks in the auxiliary work.

Finally, PTs generally support the use of prevention programs, basing their decision on the available scientific evidence. However, they believe that improvements can still be made in these processes, which contribute to the prevention of injuries.

## 5. Conclusion

This study explored the contemporary practices of PTs in professional soccer across two continents. The data suggest that the evaluation of physical capacities is based on scientific evidence and different methodologies depending on the stage of the season (pre-season, competitive period, or end of the season). It became apparent that for some stages of the season, emphasis was placed on the development and optimization of physical qualities, while optimizing recovery and fatigue management was prioritized in other stages of the season. In contrast, the PTs monitored and managed the training load, supported the use of technological tools (e.g., GPS devices), and considered this aspect a fundamental part of the role. However, they also supported the use of subjective questionnaires, since some players deviated from the team average and, as such, required bespoke intervention. Finally, the PTs recognize the use of injury prevention programs, which are multi-faceted in nature, and require the support of several disciplines working in sync. However, there was a revelation that since contextual factors can influence the adherence to strictly working within the scientific guidelines, practice must change to encourage player engagement. Therefore, although the scientific guidelines are closely followed, some flexibility is shown within the role to accommodate a multitude of contextual factors. For current and future PTs who want to enter the sports discipline, this study will be a reference of what is done in high performance and the nuances that can appear in different situations during a season.

## 6. Limitations of the study and possible future studies

The main limitations of this research arise when considering a small number of participants. In contrast, it is possible that future investigations of this type replicate this interview in other contexts, compare, deepen, or adapt certain criteria that increase the knowledge of the practices of PTs in soccer or other sports disciplines.

## Data availability statement

The original contributions presented in the study are included in the article/Supplementary material, further inquiries can be directed to the corresponding author.

## Ethics statement

The studies involving human participants were reviewed and approved by Scientific Council and the Ethics Committee of the University of Coimbra (CE/FCDEF-UC/00692021). The patients/participants provided their written informed consent to participate in this study.

## Author contributions

JB-D, AJF, and HS: project direction, established the protocol, wrote and revised the original manuscript, and final approval for publication. AF: data analysis, critical revision, and final approval for publication. BF, SQ, JS, JR, IP, PC, HT, and AS: delivery of evidence of their professional work, critical and exhaustive review of the writing of the study in general, and final approval for publication. All authors contributed to the article and approved the submitted version.
