# Supplementary material for: Contemporary practices of physical trainers in professional soccer: A qualitative study
Source: Front Psychol. 2023 Sep 19;14:1101958. doi: 10.3389/fpsyg.2023.1101958 (PMC10548828; doi:10.3389/fpsyg.2023.1101958)
Supplement: Supplementary file 1 [file Table_1.DOCX]

Interview

| **A) Assessment of physical abilities.**  1- In recent years, the scientific literature has increased considerably, classifying football as a sport " *multifactorial, where players must have well-developed physical, psychological, technical and tactical capabilities"* [1]. I would like to know if you do some kind of test to assess the physical abilities of your athletes?  1.1- If the answer is YES, for capacity... and at what time of the season? |
| --- |
| 1. For aerobic capacity?, 2. For anaerobic capacity?, 3. For the speed?, 4. For agility?, 5. For strength?, 6. For anthropometry?, 7. For coordination?, 8. Other |
| 2- How is the collected information analyzed and how is this knowledge transferred to practice (e.g., statistical analysis, average, mode, Z-score, etc.)? Can you give an example? How do you use and what impact do these results have on individual and collective planning? |
| 1. Statistical analysis (mean, Z-Score, standard deviation, classification) 2. Publications 3. Other |
| * Although football is a multifactorial sport, as it focuses exclusively on the physical abilities of athletes...  3- Do you consider that there is a physical capacity that is more important than the others?  3.1- If the answer is YES- Which one? Justify the answer affirming or denying (because of what) ... could you give an example? |
| 1. Strength 2. Speed 3. Resistance 4. Other |
| 4- Within the weekly microcycle, is there any logical order of physical skill work?  4.1- If the answer is YES- Which one? Justify the answer by affirming or denying (because of what) ... could you give an example? |
| 1. Preparatory microcycle 2. Directed transformation microcycle 3. Special transformation microcycle 4. Maintenance microcycle. 5. Competitive microcycle |
| 5- Do you want to add some information about physical capacity assessment that has not been mentioned or reinforce others? |
| 1. Yes, why? 2. No, why? |
| **B) Training monitoring and control.**  Currently, the monitoring of athletes of a high competitive level is becoming more frequent, since, "*The monitoring and control of the athlete/team helps to establish a balance between performance and recovery*" [2-4], "*in addition to guaranteeing a load of ideal work, that allows to maximize the physical condition and to prevent injuries or illnesses*" [5].  1- Do you use any method of evaluating and controlling physical performance in training and games?  If the answer is YES, can you indicate which one? |
| 1. Subjective perception of effort, 2. GPS, 3. Heart rate meters, 4. Others. |
| 2- How is the collected information analyzed and how is this knowledge transferred to practice (e.g., statistical analysis, Media, mode, Z-score, etc.)? Can you give an example? How do you use and what impact do these results have on individual and collective planning? |
|  |
| 3- Do you consider that the Perceived Exertion Scale (RPE) and the Recovery Test (TQR) are valid indicators to estimate the internal load of athletes? |
| 1. Yes, why? 2. No, why? |
| 4- Do you use another method to estimate the internal load, such as... lactate or heart rate monitors?  4.1 If the answer is YES, can you indicate how? |
|  |
| 5- In your experience, which instruments would you consider essential for today's high-performance monitoring? |
| 1. GPS, 2. Acute burden/chronic burden ratio, 3. Other. Because? |
| 6- Do you want to add some information about training monitoring and control that has not been mentioned or reinforce others? |
|  |
| **C) Injury prevention.**  Injuries are a sensitive issue for all football clubs, it has been determined that in elite clubs,*"* *each player unable to play due to an injury represents an economic cost of €20,000 per day in elite clubs, in addition to the constraints associated with the sporting part"* [6].  1- What are the risk factors that should be taken into account when trying to anticipate injuries? |
| 1. Fatigue, 2. A previous injury, 3. Age, 4. High workloads or peak workload increases, 5. Poor recovery (sleep), 6. Large number of games in a short time, 7. Player position, 8. Result or place of the game, 9. Winter vacation, 10. Psychological factors, 11. Others. |
| 2- Do you use any programs to prevent injuries? |
| 1. FIFA 11+, 2. Eccentric tendon exercises, 3. Adductor strengthening, 4. Proprioception, 5. Multicomponent, 6. Flexibility programs, etc? 7. Others. If you answered others, can you specify what it consists of? |
| 3- Achieving high player participation is critical to the success of prevention programs. In this sense, have you had difficulties in the implementation and participation of players in prevention programs or another situation? |
| 1. Yes, why? 2. No, why? 3. Other. |
| 4- Do coaches and medical staff value implementing these injury prevention programs? Can you explain how? |
| 1. Yes, why? 2. No, why? 3. Other. |
| 5- Do you think prevention programs could be improved in any way? Can you explain how? |
| 1. Yes, why? 2. No, why? |
| 6- Bearing in mind the complex sporting calendars and the understanding that each game is important. How do you design and articulate prevention programs in each microcycle? |
| 1. Yes, why? 2. No, why? |
| 7- Is the construction or design of prevention programs general, individual or mixed? Because? |
| 1. General 2. Individual 3. Mixed |
| 8- Do you want to add some information about injury prevention that wasn't mentioned or reinforce others? |
| 1. Yes, why? 2. No, why? |

**References**

1. Grendstad, H., et al., *Physical capacity, not skeletal maturity, distinguishes competitive levels in male Norwegian U14 soccer players.* Scandinavian Journal of Medicine & Science in Sports, 2020. **30**(2): p. 254-263.

2. Gaudino, P., et al., *Factors influencing perception of effort (session-RPE) during elite soccer training.* International Journal of Sports Physiology and Peformance, 2015.

3. Impellizzeri, F.M., et al., *Use of RPE-based training load in soccer.* Medicine & Science in sports & exercise, 2004. **36**(6): p. 1042-1047.

4. Moalla, W., et al., *Relationship between daily training load and psychometric status of professional soccer players.* Research in Sports Medicine, 2016. **24**(4): p. 387-394.

5. Jaspers, A., et al., *Relationships Between Training Load Indicators and Training Outcomes in Professional Soccer.* Sports Medicine, 2017. **47**(3): p. 533-544.

6. Ekstrand, J., *Preventing injuries in professional football: thinking bigger and working together.* British Journal of Sports Medicine, 2016. **50**(12): p. 709-+.
